# Perceptions of healthy eating and physical activity in an ethnically diverse sample of young children and their parents: the DEAL prevention of obesity study

**DOI:** 10.1111/j.1365-277X.2012.01280.x

**Published:** 2012-07-25

**Authors:** E Rawlins, G Baker, M Maynard, S Harding

**Affiliations:** * School of Social and Community Medicine, University of BristolBristol, UK; † Moray House School of Education, University of EdinburghEdinburgh, UK; ‡ Medical Research Council/Chief Scientist Office, Social and Public Health Sciences UnitGlasgow, UK

**Keywords:** ethnic minorities, families, interventions, obesity, qualitative, UK

## Abstract

**Background:**

Ethnicity is a consistent correlate of obesity; however, little is known about the perceptions and beliefs that may influence engagement with obesity prevention programmes among ethnic minority children. Barriers to (and facilitators of) healthy lifestyles were examined in the qualitative arm of the London (UK) DiEt and Active Living (DEAL) study.

**Methods:**

Children aged 8–13 years and their parents, from diverse ethnic groups, were recruited through schools and through places of worship. Thirteen focus group sessions were held with 70 children (*n* = 39 girls) and eight focus groups and five interviews with 43 parents (*n* = 34 mothers).

**Results:**

Across ethnic groups, dislike of school meals, lack of knowledge of physical activity guidelines for children and negativity towards physical education at school among girls, potentially hindered healthy living. Issues relating to families' wider neighbourhoods (e.g. fast food outlets; lack of safety) illustrated child and parental concerns that environments could thwart intentions for healthy eating and activity. By contrast, there was general awareness of key dietary messages and an emphasis on dietary variety and balance. For ethnic minorities, places of worship were key focal points for social support. Discourse around the retention of traditional practices, family roles and responsibilities, and religion highlighted both potential facilitators (e.g. the importance of family meals) and barriers (reliance on convenience stores for traditional foods). Socio-economic circumstances intersected with key themes, within and between ethnic groups.

**Conclusions:**

Several barriers to (and facilitators of) healthy lifestyles were common across ethnic groups. Diversity of cultural frameworks not only were more nuanced, but also shaped lifestyles for minority children.

## Introduction

Ethnicity is a consistent correlate of obesity in childhood (Wardle *et al*., 2006; Harding *et al*., 2010). In adolescence, Black African and Black Caribbean boys and girls are more likely to be overweight than their White peers, and South Asian origin children have a greater propensity to central adiposity (Whincup *et al*., 2002; Harding *et al*., 2010). Co-morbidities such as type 2 diabetes, hypertension and cerebrovascular disease are more common among adult Black African populations (Cruickshank *et al*., 2001; Chaturvedi, 2003), and a potential worsening of obesity-related risk in their children carries implications for persisting disparities in chronic disease across generations. Tackling rising childhood obesity levels has been identified as a government policy priority (Zaninotto *et al*., 2006; Department of Health, 2008). Concerns over rising health care costs associated with the potential development of co-morbidities such as heart disease and type 2 diabetes, as well as adverse psychosocial outcomes in adolescence or early adulthood (National Audit Office, 2001; Lobstein *et al*., 2004), have furthered interest in the aetiology of childhood obesity.

Both the prevalence and the social patterning of childhood obesity have changed rapidly, suggesting that adverse environmental factors rather than genetic factors are the primary cause (Law *et al*., 2007). Lower levels of obesity among West African populations in West Africa than in the Caribbean and the USA supports the hypothesis that environmental influences (e.g. changes in diet and activity habits) explain the increased risk of obesity among those migrating from low to high income countries (Cruickshank *et al*., 2001). Migration may be accompanied by a shift from traditional diets to dietary patterns more common in the host population among some ethnic groups. A comparison of UK-born Caribbean and migrant Caribbean adults indicated a shift from traditional cuisine rich in fruit and vegetables to a diet higher in fat intake (Sharma *et al*., 2002). Dietary acculturation among children of migrants is probable because they are more likely to engage in local food cultures than their parents (Gilbert & Khokhar, 2008).

There are few UK-based studies on ethnic differences in obesity promoting behaviours in children. In a UK study of adolescents, some ethnic minorities appeared to engage more than their White British peers in dietary practices that promote obesity, particularly among those born in the UK than those born abroad. For example, compared to White British girls, Black Caribbeans, Black Africans and Pakistani girls were more likely to skip breakfast and consume sweetened carbonated drinks daily and less likely to have the recommended daily intake of fruit and vegetables (Harding *et al*., 2008). Among primary school children, Black Caribbeans consumed more saturated fats than Black African children (Donin *et al*., 2010), although both Black African and Black Caribbean children were less likely to be sedentary than their White British peers (Owen *et al*., 2009).

Interventions involving the whole family rather than just the individual are supported in the obesity treatment literature (Golan & Weizman, 2001) because social support from family members may encourage healthy dietary adherence (Wilson & Ampey-Thornhill, 2001). Ethnic-specific health intervention studies in the USA have often involved promoting social support from both the family and community, as well as obtaining direct access to specific populations (Fitzgibbon *et al*., 2002; Beech *et al*., 2003). The importance of family and community values (religion, community, kinship and cultural heritage) and identities over individualistic behaviour has been a particularly strong theme in research relating to African and South Asian cultures (Maiter & George, 2003; Barn *et al*., 2006). For ethnic minority children whose family practices remain more closely aligned with the country of origin, there is the potential for contradictions with practices encountered outside the home (Berry *et al*., 2006).

The overall aim of the DiEt and Active Living (DEAL) study was to identify culturally acceptable child- and family-based interventions aiming to reduce dietary and physical activity risk factors for childhood obesity among ethnic minorities, in both schools and places of worship. We explored how potential interventions might work and the setting most likely to support effective engagement. DEAL draws on the principles of the Medical Research Council framework for development and evaluation of complex interventions, which advocates small scale development and feasibility/piloting phases (Craig *et al*., 2008). One aspect of the DEAL study was the qualitative exploration of the barriers to (and facilitators of) healthy eating and activity habits in ethnic minority children. Focus groups and interviews were therefore conducted in this arm of the study to explore, in some depth, both individual and family perceptions, intentions and beliefs relating to healthy lifestyles. We also aimed to engage with how participants' viewed their intentions to be shaped by wider contextual influences (e.g. food outlets and outdoor spaces), informed by a socio-ecological model (Stokols, 1992). Additionally, although parents may be the main mediators in negotiating their children's health, children are active agents in engaging (or not) with healthy lifestyle practices. To facilitate connecting with children's own views, we conducted child-only focus groups [informed by the ‘health-promoting family’ model developed by Christensen (2004)]. In the present study, we report on the findings from these focus groups and interviews with children and parents.

## Materials and methods

### The DiEt and Active Living study

Black Caribbean, Black African, Indian, Pakistani, Bangladeshi and White British children and their parents were recruited through schools or places of worship. Three primary schools and two secondary schools participated in the present study. This included one Church of England and one Pentecostal church (providing access to Black African and Black Caribbean families), one mosque (Pakistani and Bangladeshi families), one Hindu temple, one Tamil temple and one Sikh Gurdwara (Indian families). These were located in the London boroughs of Brent, Croydon, Ealing, Hackney, Hillingdon and Lambeth. Pupils in the first year (aged 11–12 years) at two secondary schools and in the final year (aged 10–11 years) of three primary schools were recruited from the five specified ethnic minority groups along with White UK pupils. These ages were selected as an important period of transition in young people's lives that could require different behaviour modification strategies. Potential participants in schools were selected by key contacts (teachers and head teachers) (Maynard *et al*., 2009). In places of worship, the age range was extended (8–13 years) because it was difficult to refuse children who were very enthusiastic to join in. Children and parents in places of worship responded to recruitment drives, were present on the day or entered the study via snowball recruitment (Maynard *et al*., 2009). All participants completed a screening questionnaire to ensure broad representation by age, gender and socio-economic status (parental education level, occupation, employment) within ethnic groups. Approval for the study was obtained from the University of Glasgow's Medical Faculty Ethics Committee, as well as relevant religious organisations.

Ethnicity was self-defined using a 20-option grid, based on ethnic categories from the 2001 UK Census. Children who reported their ethnicity as ‘Black British’, or who did not report their own ethnicity, were classified by parental ethnicity and country of birth. Where children reported an ethnicity inconsistent with parental ethnicity, parental ethnicity was used as a substitute. If data were missing for parents, or for both parents and children, ethnicity was ascribed by the researcher if this was alluded to during focus groups or interviews (e.g. the parent stated that s/he was born in the Caribbean) and in consultation with teachers. Using The National Statistics Socio-economic Classification (NS-SEC), parents in work were classified to an occupational class (non-manual/professional, intermediate jobs and partly skilled or unskilled manual jobs). If not in work, they were classified as looking after the home and family or unemployed. Parental education was coded as having completed secondary/high school or further education (vocational qualification/college or university educated). For both occupation and education, father's status was given priority and mother's details used only if the father's details were missing. Low SEC in the present study generally refers to parent classified as manual and high SEC to non-manual.

### Focus groups and interviews

The aim of the focus groups and interviews was to elicit perceptions, intentions and beliefs relating to barriers to and facilitators of eating a healthy diet and participating in physical activity (PA). Discussion topics included health related knowledge and attitudes, preferences for diet and activity patterns (at school and elsewhere), food shopping, eating out, physical education lessons, PA facilities, and family life. Probing questions were utilised where necessary on what helped and hindered participants' in making healthy choices in each area (e.g. cost, access, likes and dislikes, competing priorities). Development of the topic guides was aided by existing literature on the theoretical frameworks that informed the study (Stokols, 1992; Christensen, 2004), and previous qualitative research on healthy lifestyles among children from diverse ethnic groups (Rawlins, 2008). Pilot focus groups were carried out with pupils from one of the study schools, enabling refinement of the topic guide. During the piloting phase, it was also established that the topic guides encouraged discussion of personal experience and perceptions of healthy lifestyles. The appropriateness of ‘ice breaker’ tasks such as pen and paper exercises and games was also tested in the pilot phase. The finalised topic guides are available on the DEAL website (http://dash.sphsu.mrc.ac.uk/public/DEAL/).

Following the piloting stage, focus groups and interviews took place during September 2008 to June 2009. Focus groups were conducted with either children or parents. Individual semi- structured interviews were conducted with parents where organising focus groups proved difficult as a result of shift-working patterns. The focus groups with children were conducted for approximately 45 min and group discussion and interviews with adults for approximately 1 h. All interviews and focus groups were conducted in English, facilitated by the first author who is female and of White British ethnicity. All discussions were recorded, with the participants' consent, and were transcribed verbatim. The qualitative software package nvivo (version 8; QSR International Pty Ltd, Melbourne, Australia) was used to assist data management and analysis. All authors independently coded the transcripts and met regularly to discuss and refine the themes in relation to theoretically driven concepts. The data were examined within and between ethnic groups by gender [denoted in text by male (M) and female (F)], parental country of birth, and parental SEC to identify both general and ethnic specific influences and how these intersected with SEC. The codes attached to the quotes reflect these categories in the order of gender, age of child or parent, parental country of birth, parental education, and parental occupation. For example, an 8-year-old Black Caribbean boy whose parent was born in the UK, educated beyond secondary school, and was classified to a manual class, was coded as: Child: Black Caribbean, M, 8; Parent: UK-born, further education, manual. Labels that do not contain all of this information reflect missing data.

## Results

Table 1 shows the sample characteristics. In total, 70 children (56% girls) aged 8–13 years and 43 parents (79% mothers) took part in the qualitative aspect of the DEAL study. In schools, almost twice the number of girls were recruited than boys as a result of the inclusion of an all-girl secondary school. Eighteen children were classified by parental ethnicity and country of birth. Ethnicity was assigned by the researcher for eight parents and seven children. Table 2 shows the distribution of parental characteristics for the 70 children who took part in DEAL, by ethnicity. More Pakistani and Bangladeshi children, as well as both their parents, were born abroad than in any of the other groups. Black Caribbean and Black African mothers were more likely to be in non-manual occupations, whereas Indian, Pakistani and Bangladeshi mothers were more likely to be doing full-time housework than mothers from other ethnic groups. Thirteen focus group sessions were held with children and eight focus groups and five interviews with parents (Table 3).

**Table 1:** DEAL study sample characteristics: number of participants by setting, gender and ethnic group

	Children			Parents		
	
	Boys	Girls	All	Mothers	Fathers	All
Total sample	31	39	70	34	9	43
*By setting*						
School	11	21	32	10	4	14
Place of worship	20	18	38	24	5	29
*By ethnic group*						
Black Caribbean	4	3	7	5	0	5
Black African	3	7	10	5	1	6
Indian	11	13	24	15	4	19
Pakistani	5	6	11	3	2	5
Bangladeshi	3	3	6	2	1	3
White UK	2	3	5	1	1	2
Other*	3	4	7	3	0	3

* Includes mixed ethnicity.

**Table 2:** DEAL study sample characteristics: distribution of parental characteristics for children who took part in DEAL*, by ethnicity

	Black Caribbean (*n* = 7) (%)	Black African (*n* = 10) (%)	Indian (*n* = 24) (%)	Pakistani (*n* = 11) (%)	Bangladeshi (*n* = 6) (%)	White UK (*n* = 5) (%)	Other (*n* = 7) (%)
Father – non-manual occupation	43	45	48	45	33	40	57
Mother – non-manual occupation	57	64	21	9	0	20	57
Mother – full-time housework	0	18	34	45	100	20	0
Two-parent family	43	73	97	100	100	60	100
Child born abroad	0	9	10	36	33	0	0
Both parents born abroad	0	45	18	54	50	20	14

* Parental characteristics were obtained for all 70 children who took part in DEAL, even though only 43 parents took part in interviews themselves. The information was obtained from screening questionnaires at the start of the study.

**Table 3:** Number of focus groups/interviews conducted, by location

	Number of focus groups/interviews (number of participants in each event)	
	
	Children	Parents
Primary schools	3 (8: 7: 3)	4 (2: 3: 1: 1)
Secondary schools	3 (6: 4: 4)	3 (5: 1:1)
Churches	2 (5: 3)	2 (5: 4)
Mosques	2 (6: 6)	1 (6)
Sikh Gurdwara	1 (4)	1 (1)
Hindu temple	1 (7)	1 (3)
Tamil Hindu centre	1 (7)	1 (10)
Total number of focus groups/interviews	13	13

The themes and sub-themes emerging from the data, categorised as potential barriers to (and facilitators of) healthy lifestyles are shown in Table 4. A narrative presentation of the data supported by quotes is given below. Common themes are first reported before focussing on what appeared to be ethnic specific influences. This dichotomy was not always clear cut and reflected the subtle interplay between ethnic specific and more common themes.

**Table 4:** Themes emerging from the analysis of the focus groups and interviews and potential barriers to and facilitators of healthy lifestyles

Main themes	Sub-themes	

	Healthy lifestyle barriers	Healthy lifestyle facilitators
*Influences common to all ethnic groups*		
1. Interpretation of diet and health promotion messages		
Children	Views on nutritional adequacy of foods and on a balanced diet were at odds with conventional definitions	–
Children and parents	–	General awareness of health promotion messages
Parents	Belief that some health promotion messages are poorly conveyed	–
2. Dislike of school meals		
Children	Resistance to improvements to the quality of school meals, and concern about hygiene practices	–
3. SEC influences on shopping practices		
Children	–	–
Children and parents	–	
Parents	Low SEC more reliant on budget supermarkets, ‘corner shops’, convenience stores and ‘shopping around’	High SEC shop in large supermarkets
4. Physical activity preferences and knowledge		
Children	Lack of interest in physical education classes among girls;	–
Children and parents	Concern about the cost	Variety of family activities
Parents	Limited awareness of current physical activity recommendations for children	Discussion of activities families would like to do if affordable
5. Neighbourhood constraints		
Children	–	–
Children and parents	‘Stranger danger’ fears in outside play space; – gangs, unsafe dogs and violent attacks among low SEC and road safety among high SEC	–
Parents	Fast food outlets thwarting good intentions; low SEC report a lack of physical activity facilities	–
*Ethnic specific issues*		
6. Retention of traditional practice		
Children	–	–
Children and parents	Regular family meals – Indian families: – Meal time discussion highlighted the issue of homework being more important than other activity (e.g. physical activity)	Regular family meals – opportunities to: – Monitor eating habits – Discuss wider issues of general wellbeing
Parents	Obtaining Black African and Caribbean foods – Requires shopping around – Reliance on convenience shops (high prices and possibly poorer quality)	Obtaining South Asian foods – available in mainstream shops reducing the need to shop around Obtaining Black African and Caribbean foods – Black Africans and Black Caribbeans also frequenting markets (reasonably priced, fresher produce)
7. Roles and responsibilities of family members		
Children	Gender specific roles regarding shopping and food preparation	–
Children and parents	Black Caribbeans and Black Africans – matriarchal households, low participation of men in main food preparation All minority groups: – role of convenience food and fast food outlets accommodating ethnic preferences	Extended family members an important presence in homes or nearby
Parents	–	Attitudes towards male participation in food related chores changing
8. Religion		
Children	–	Involvement in checking food labels for proscribed foods (particularly where literacy was an issue for parents)
Children and parents	–	–
Parents	Variable promotion of physical activity in places of worship	Places of worship support for promotion of health messages relating to diet

SEC, socio-economic circumstances, based on the National Statistics Socio-economic Classification. Low SEC refers to mothers classified as manual and high to those classified as non-manual.

### Influences common to all ethnic groups

#### Interpretation of diet and health promotion messages

Reports on how healthy eating could be achieved reflected children's own creative interpretations about the nutritional adequacy of foods. An 8-year-old boy stated that he drank ‘Fruit Shoot [premixed juice drink] because it's fruit’ (Child: Black Caribbean, M, 8; Parent: Black Caribbean, UK-born, further education, manual), whereas another said he ate ‘healthy’ Cheese Strings [processed cheese that can be torn in strips or ‘strings’] ‘Cos they have milk in it’ (Child: White British, M, 11; Parent: White British, UK-born, further education). Almost all children had heard of ‘food groups’ during Personal and Social Health Education or food technology lessons but found they could not specifically recall the main food groups (fruits and vegetables; starchy foods; meat, fish, beans and eggs; milk and dairy products; foods containing high fat and sugar).

Children's views on a balanced diet were at odds with formal definitions: a balance of required macro and micronutrients from a variety of foods from the five main food groups; low in fat (especially saturated fat), salt and sugar (Food Standards Agency, 2001). Children's interpretations of ‘balance’ centred around ‘healthy’ and ‘unhealthy’ foods being eaten in equal measure. As this girl explains:

‘Like if you want sweets you have the same amount of fruit as well, so you can combine’ (Child: Black African, F, 11; Parent: Black African, Ghana-born, further education)

Despite limitations in knowledge there was, however, a general awareness of key dietary messages (e.g. eating more fruit and vegetables, cutting down on sugary and fatty foods). Parents were aware of the need to eat a balanced diet but felt that the concept of a balanced diet or lifestyle was not well conveyed, as this father states:

‘Everybody talks about sort of having a balanced lifestyle. That means a balanced meal, and balanced exercise. But the problem is we don't actually know what the balance is' (Father: Indian, 46, Kenya-born, further education, non-manual)

Parents highlighted the importance of eating a variety of foods, as this mother explains ‘on your plate, or during the course of a day there should be a choice of things on there’ (Mother: White British, 43, UK-born, secondary school, non-manual). Other parents emphasised not overindulging in any area of food choice. Therefore, although parents felt that they lacked technical knowledge, their lay explanations of variety and not overindulging guided their approach to a healthy diet.

#### Dislike of school meals

The degree to which school meals were disliked emerged as a potentially significant barrier to children obtaining a healthy meal during the school day. There was a general feeling that statutory efforts to improve the nutritional quality of school meals had led to certain foods being replaced with other, less enjoyable, foods.

The overcooking of meals, issues of taste and the visual aspects of foods were also mentioned several times; for example, foods that were of the ‘wrong colour’, ‘mouldy’ or perceived to be ‘leftovers’ were deemed unpalatable and perhaps even ‘past their best’. The palatable attributes of foods also extended beyond the look and the taste of the foods to include how they were prepared and served, as this 10-year-old boy complains about hygiene practices:

‘Yeah, like, cause they have glove – no sometimes they don't. I was getting pasta and um, then there was some of it falling off and they just put their hand there and put it all on my plate’ (Child: Black Caribbean, M,10; Parent: Black Caribbean, UK-born, further education, non-manual)

#### Socio-economic classification influences on shopping practices

Family shopping practices differed by SEC with higher SEC mothers more likely to shop at supermarkets or via the internet to avoid the ‘hassle’ of fitting the shopping into an already busy schedule. Lower SEC families mentioned shopping in budget supermarkets such as Iceland (which specialises in frozen foods), the local markets or buying a small number of items in corner shops to avoid overspending**,** as this mother states:

‘If you go to the corner shop just down the road, you know exactly, that I want to go for bread, I will be coming out with the bread. You're not diverted, no’ (Mother: Black Caribbean, 50, Antigua-born, further education, manual)

Despite leading busy lives, the importance of economic constraints had over-ridden the ease of shopping in one place for many low SEC families, as one mother commented ‘we have to, like, shop around to get the cheapest’ (Mother: Bangladeshi, Bangladesh-born, secondary school, housework).

#### Physical activity preferences and knowledge

Although girls did not explicitly report disliking physical activity, they gave reasons for not enjoying physical education lessons at school. For example, a lack of confidence in their competence in sports, especially when participating in team games because ‘only the best get chosen and then if you're not very good it's, like, you're not wanted’ (Child; Mixed ethnicity, F, 11). Many of these concerns over sporting ability were also discussed in relation to mixed gender games and the differences between boys' and girls' abilities. The attitude of boys was a hindrance to PA participation, as mentioned on several occasions; either the boys did not wish to engage with girls or hogged the equipment so the girls could not use it.

Parents had very limited knowledge of UK PA recommendations for children [currently at least 1 h of moderate to vigorous physical activity every day (Department of Health, 2004)]. As this mother commented ‘I know about the recommended food, but not for exercising kids’ (Mother: Black Caribbean, UK, further education, non-manual). The discourse on physical activity generally lacked detail or enthusiasm. By contrast to conversations about food (however sparse in detail), those about physical activity were delivered in much less assured terms. The discussion around physical activity practices and knowledge of recommendations was much more succinct. Guesses at the amount of recommended physical activity for children varied between 10 min to several hours.

Both children and parents did, however, give some description of activities done as a family. In the children's discourse, there was also an element of nostalgia for family when they were younger. Among parents, there was an emphasis on family activities they would like to do in the future. For the lower SEC parents, cost was cited as a significant barrier. Swimming was mentioned regularly, with families utilising free swimming sessions where available. The loss of free swimming sessions for children could have a significant impact on their activity levels, as this parent indicates:

‘Well this, there are cost implications obviously. I mean if I did, I were to go swimming, and to take all three of my kids with me, it'd cost me about twelve pounds. For an hour’ (Father, Indian, 46, Kenya-born, further education)

#### Neighbourhood constraints

The issue of ‘stranger danger’ was mentioned many times by both parents and children in discussions about what stopped them from using local park facilities or why children were not allowed out unsupervised. In many cases, issues about older children and other strangers in the local parks focused on particularly violent examples of safety concerns. As this 11-year-old girl complains ‘There's too many dogs […] and too many gangs as well hanging around there. And when you go in there you can just get shot' (Child: Black African, F, 11; Parent: Black African, Ghana-born, further education). Concerns around road safety were also cited as a reason why children could not play outside unaccompanied, walk home by themselves or ride their bikes or scooters in the local area. Parents and children in higher SEC groups appeared to be more concerned with road safety, whereas those from lower SEC groups were more concerned with dangers posed by strangers, gangs, unsafe dogs and violent attacks. Overlapping with the theme of physical activity preferences, parental and child concerns regarding an absence of PA facilities, or poor quality of existing facilities, was evident.

One of the most common concerns for parents, especially of older children, was the availability of ‘junk’ foods outside the home. They felt that the proliferation of fast food outlets around schools and their homes thwarted their efforts to ensure that their children ate healthily, particularly because of the low cost.

‘It's not easy because there are so many fast food shops and most of them are selling their food for one pound […] so you know you really got to stipulate to the child, this is what I would like you to eat because, it's healthy for you, you know they will duck and dive behind your back’ (Mother: Black Caribbean, 50, Antigua-born, further education, manual)

In recognising attempts by children to ‘duck and dive’ behind parent's backs, there was a sense of inevitability of children eating fast foods, so even if parents spoke to children about what was healthy and restricted their pocket money the children would still find a way to access unhealthy foods.

### Ethnic specific influences

#### Retention of traditional practices

The importance of preserving traditional customs was clear in the discourse on family meals and obtaining the necessary foods for culture specific cuisine. Regular family meals were seen as an ideal eating practice for many of the ethnic minority families in the present study, where food choices could be monitored and healthy choices facilitated. Parents were generally aware that children could resist eating certain foods and reiterated the importance of parental responsibility in encouraging children to eat healthier foods ‘until they realise this is good for them’ (Father: Indian, 42, India-born, higher education, non-manual).

Family meal times were also a chance for families to discuss wellbeing in more general terms, encompassing issues such as emotional wellbeing.

‘[…] And we stay as a family and we'll talk about how our day was, what would we like to do in the weekend’ (Child: Black African, M, 10; Parent: Black African, Nigeria-born higher education, non-manual)

For South Asian families, there was a clear sense that family meals also provided an opportunity to discuss the everyday practices of family life. In particular, when discussing which activities were most popular, it was clear that activities such as homework were a priority for some families. This was commonly mentioned by Indian children and their parents regardless of SEC. Parents felt that their children, especially when studying for examinations, had a heavy workload. The importance of doing well was not only an educational achievement in itself, but also related to potential earning power in the future. The idea of making the most of opportunities available to do better than one's parents was prevalent, as this father explains:

‘I don't know, but like, you know, for our community, you know, I would like my children to do study first […] But like, our mentality, that the way I am working, I would like to work, you know, same way my son, because I'm doing the donkey work, be honest with you, and I wouldn't like my son do the same thing’ (Father: Indian, 42, India-born, higher education, manual)

Obtaining foodstuffs for traditional meals revealed more nuanced experiences of ethnic minority parents with regard to shopping practices. For the South Asian groups, it was felt that many of the high street supermarkets sold traditional South Asian cooking ingredients, reducing the need to shop around. By contrast, Black Caribbean and Black African groups felt hindered by a lack of their traditional foods available in the high street supermarkets and they tended to shop in ‘the African shop’ or the market because ‘the market caters for everyone’ (Mother, 50, Black Caribbean, Antigua-born, Further Education, manual). This meant that those on low incomes were more likely to shop in places offering ‘traditional’ foods, potentially enabling them to maintain cultural practices if, as mentioned by many parents, ‘you have the knowledge and skills to cook’.

#### Roles and responsibilities of family members

Different family contexts revealed different roles and responsibilities for family members when it came to the practices of everyday life such as preparing and eating meals, and determining leisure time activities. In terms of food practices, women were generally in charge of shopping for (and preparing) food. For those women who also worked outside the home, food practices exemplified the competing priorities of home and work. For example, some women described rising very early in the morning to begin the preparation of the evening meal, before setting off for work. Lower rates of male participation and involvement were mentioned more often by low SEC South Asian families reflecting demarcated gender roles relating to food preparation and associated chores. This was often explained as a cultural tradition, something that children accepted as ‘the way things are’ as the following dialogue demonstrates:

‘Because in our culture all the girls have to when they get married –

Yeah –

No, when they have to, um get married.

When they marry they have to make their husbands their food forever. The woman has to cook. They have to learn' (Children in a temple: Indian ethnicity, boys and girls aged 8–11)

The idea that ‘women's work’ involved housework and ‘men's work’ involved wage earning outside the home was regularly put forward, especially by low SEC families, and those born abroad. Fathers appeared to play a minimal role in monitoring their children's eating habits because of the burden of employment; however, there were signs of opinions changing as some women drew a distinction between life ‘back home’ and here in the UK, as the following demonstrates:

‘[it's a] tradition as I said, because here, if you go back home India, when you become like all girls become ripe, start from teen age, they have to, you know, participate in cooking. Because the parents think when they get married, they need to know, they have to be best cooker. So they have to learn' (Father, 42, India, India-born, Higher Education, manual)

‘Back home, yeah’ (Mother, 40, Indian, India-born, Higher Education, manual)

There was some evidence of fathers being involved with providing meals, although these incidences were regarded as a treat or a break from the mother's cooking. As this Indian mother explains, ‘always men cooks the best [*laughter*] so children sometime wants the best cook. He cooks really good' (Mother, Indian, Indian-born). The tension between the strong desire to maintain traditional practices, children's resistance and competing priorities was negotiated in part by the use of culturally tailored convenience foods such as Halal chicken nuggets and burgers. Fast food chains (of increasing concern to parents, see above) further compounded this issue with the accommodation of ethnic preferences (e.g. Halal franchises of Fried Chicken shops).

There were different family routines in Black Caribbean and Black African families, with a more matriarchal-style family structure being common among the participants. Fathers were rarely mentioned when discussing everyday family practices, even when the father was resident in the household. With relation to cooking practices, fathers were mentioned in relation to treats of either traditional or nontraditional foods, often linked to fast foods or takeaways, as this quote illustrates:

‘My dad, sometimes if he – he cooks ackee and saltfish but sometimes if he doesn't cook breakfast he just take us to McDonalds to get pancakes’ (Child: M, 10, Black Caribbean, Parent: Black Caribbean, UK-born, further education, non-manual)

Different family types were also noticeable in the form of extended families with networks of informal support extending beyond the nuclear family. Several of the South Asian children had grandparents living with them as well as other family members nearby. In the Black Caribbean groups, there were mentions of aunts or uncles living in close proximity who also played a role in childcare and family life more generally. Definitions of ‘family’ across these groups clearly went beyond those living in the family home.

#### Religion

Intersecting with issues around ethnic specific shopping practices, checking for proscribed ingredients on food labels among those eating a religious diet (e.g. gelatine by Hindus; non-Halal meat/meat products by Muslims) was a necessary custom. Several of the participants mentioned different strategies for checking the label, including checking ingredients on the computer, as well as carrying laminated cards with the details of E-numbers and other animal-derived additives. Many of the children were involved in shopping with their parents and, as this 10-year-old boy explains, he is involved with finding Halal foods:

‘My mum takes me to the shops because she can't read English, that's why I have to go with her and to see bacon, like if it is bacon or not’ (Child: M, 10, Bangladeshi; Parent: Bangladesh, Bangladeshi-born, manual)

When asked in more detail about what else may be on food labels children and parents were aware of the other dietary information present, although this was not their main priority. This raises the possibility of exploiting a practice already in place to encourage families to use food labels in a more general manner to ensure a religious but also nutritionally adequate, diet.

During the study, it became clear that, beyond providing religious support, the places of worship attended by the participants were also a focal point for the local community. The element of social support found in the places of worship not only included formal aspects, such as religious schooling for children and young people and women's groups, but also informal support through spending time socialising, including eating together. Hindu temples and Sikh Gurdwaras regularly provided free meals, creating a way to continue traditional eating practices, as well as an informal space for members of the same religious/ethnic communities to spend time together. The Temples were a lively hub of activities for families, especially on the weekends as this Indian Hindu father explains:

‘Like, you know, all religious person like, you know, who you reckon […] temple every weekend, they are not like a westernised, you know? They'll believe in community […] So they'll always go with family […] Always together, like I have to come here, then my whole family comes. He'll settle down, he's happy to eat whatever the temple provides’ (Father, 42, Indian, India-born, Higher Education, manual)

Mosques and churches were less likely to provide regular meals but provided a space for community members to meet outside observance times. More generally, however, there was a sense that the connection between healthy diets and religion was stronger than the connection between physical activity and religion across the ethnic groups. Promoting physical activity was variable across the different places of worship, either via links with community clubs or initiatives of individuals. Ethnic or cultural specific issues were found to be more commonly reported during focus groups in places of worship.

## Discussion

The findings of our qualitative analysis of what may promote or hinder healthy living identified a number of themes common across groups, irrespective of ethnicity. Retention of traditional practices, differences in the notion of families, and the importance of religion in retaining cultural food practices were key ethnic specific themes. The interplay between themes, particularly the intersection with socio-economic circumstances, meant that ethnic specific themes were subtly nuanced.

Dislike of school meals, negativity towards physical education at school among girls, and lack of knowledge of physical activity guidelines for children were potential barriers to healthy living across groups. The girls' poor engagement with physical activity and explanations of their dislike for physical education were parallel with other studies (Mulvihill *et al*., 2000; Thompson *et al*., 2003; Rees *et al*., 2006; Jansen *et al*., 2010). The general themes in DEAL are also consistent with findings from the developmental stages of US obesity prevention studies (Thompson *et al*., 2003; Resnicow *et al*., 2005), as well as other ethnic specific qualitative research (Burnet *et al*., 2007), such as limitations in knowledge, confusion over key health messages and children's access to fast food items despite parental rules or attempts at control.

Our findings on neighbourhood constraints (e.g. fast food outlets; lack of safety) are consistent with quantitative examination of how local environments might promote or restrict healthy behaviours. Research has shown that grocery stores that sell healthy foods, safe parks and recreational facilities may promote healthy eating and physical activity (Ellaway & Macintyre, 2001). Deprived areas, which are less well served with facilities, and perceptions of safety and attractiveness of neighbourhoods, as well as the proliferation of fast food outlets, have been shown to influence eating and physical activity behaviours in surveys and qualitative studies (Rogers *et al*., 1997; Mulvihill *et al*., 2000; Rees *et al*., 2001; Parkes & Kearns, 2006). DEAL demonstrated that take-away food shops accommodating ethnic preferences, dietary acculturation, and the role of fathers in food provision, increases the complexity of the role of fast food in the diets of ethnic minorities. Strategies that engage with local councils and food retailers are clearly needed. We focused on the cultural contexts shaping people's interaction with their local environments. Nonetheless, DEAL findings lend support to a holistic, multi-level, multi-sectoral approaches to tackle the factors that influence health inequalities including childhood obesity (Bagwell & Doff, 2009; Department of Health, 2010; Ochieng, 2011).

The general facilitators of healthy living identified were fewer but included awareness of key dietary messages among children and parents. Concepts of dietary variety and balance were evident among parents, although the parents themselves felt messages about how to achieve a healthy diet were poorly conveyed.

For ethnic minorities, places of worship were key focal points for social support. Within ethnic specific themes, potential barriers such as reliance on convenience stores for obtaining traditional foods for some groups and the low priority of PA for others were evident. A number of positive factors were also emerged. Regular family meals were seen as an ideal eating practice for many of the ethnic minority families in the present study. Eating together as a family reinforces rituals and traditions and has been associated with better quality diets (Videon & Manning, 2003) and mental wellbeing (Maynard & Harding, 2010) in young people from diverse ethnic groups. Promotion of healthy traditional cuisine, using markets to obtain fresh produce and extending the existing interest in (and use of) food labels are also useful practical strategies supported by the findings.

The potential for places of worship to act as settings in culturally tailored or targeted obesity interventions in children is under-researched (Resnicow *et al*., 2005). Our finding that such settings may facilitate discussion of ethnic or cultural specific issues may be a result of the homogeneity of ethnicity in these groups, in contrast to heterogeneous school-based sessions. Alternatively, it could be that these issues are more relevant in the places of worship setting where there is greater family involvement.

This is the first UK study of its kind incorporating the interaction between families and their places of worship in exploring what facilitates and hinders healthy lifestyles among ethnic minority children. Using qualitative methods enabled us to explore the subtle interactions between ethnicity, SEC, neighbourhoods, places of worship, social networks and everyday acculturative practices. There are limitations to the study, however, that warrant mention. Focus groups are acknowledged as valuable tools in research with children, although their use raises a number of issues. Greater cohesiveness within the groups and preparedness to speak are assumed to be enhanced by the use of ‘warm-up’ sessions, and by breaking up the focus group period with further activities and refreshments. However, initial piloting of warm-up techniques and breaks in the discussion led to a lack of concentration in several of the participants. Small numbers as a result of last minute cancellations were common in some of the DEAL focus groups, as has been found in other studies (Morgan *et al*., 2002), which placed additional burden on the participants. Pen and paper exercises and games are recommended to maintain interest and minimise fatigue (Morgan *et al*., 2002) and were tested also during the piloting stages. The use of these methods yielded no additional benefits and were time consuming. Another noticeable feature of the focus groups was a tendency for children wanting to speak with the facilitator rather than with other children. Further time to develop more appropriate participatory ‘ice breaking’ tasks and strategies to promote a discourse with other participants would be useful. Among parents, during conversations around food and nutrition, there was a feeling that parents were attempting to justify their dietary habits and creating the impression of an acceptable ‘public’ narrative (Cornwell, 1984). Also relating to obtaining unbiased accounts in research, Adamson and Donovan pose the question ‘Can researchers legitimately conduct interpretive research with different-ethnicity informants?’ (Adamson & Donovan, 2002: 317). The impact of the ethnicity of the facilitator on the extent and type of data obtained in the DEAL focus groups cannot be assessed directly and there remains the possibility that the depth of views associated with ‘insider’ ethnicity were not elicited (Ochieng, 2010). Alternatively, the perceived benefits of participants' social distance from researchers (e.g. confidentiality) have also been identified (Adamson & Donovan, 2002). We feel that the minority ethnic identities of two of the authors (MM – Black Caribbean; SH – Indo Caribbean), together with the critical capacities and sensitivity of all the authors, mitigates against any concern of ethno-centric interpretation of the findings. Ethnicity is only one aspect of identity that may impact on the research process. Only 21% of the parental sample were men and ways of increasing engagement of men in research focusing on families needs further attention.

The findings of our qualitative analysis of what may promote or hinder healthy living choices indicated that obesity prevention interventions need to incorporate mechanisms for accommodating the diversity in cultural frameworks that guide everyday life. Place of worship settings may facilitate discussion of ethnic or cultural specific issues relevant to health promotion. Further research is required to fully establish the extent to which the ethnic specific themes found in DEAL may contribute to the development of obesity prevention interventions in ethnic minority children because little evidence exists to support or contrast with these issues. Greater promotion of physical activity recommendations, further exploration of engaging girls in physical activity, and integrated health and built environment policies would benefit all ethnic groups.
